# Store-operated Ca^2+^ Entry Facilitates the Lipopolysaccharide-induced Cyclooxygenase-2 Expression in Gastric Cancer Cells

**DOI:** 10.1038/s41598-017-12648-1

**Published:** 2017-10-16

**Authors:** Jhen-Hong Wong, Kuo-Hao Ho, Sean Nam, Wen-Li Hsu, Chia-Hsien Lin, Che-Mai Chang, Jaw-Yuan Wang, Wei-Chiao Chang

**Affiliations:** 1 Department of Clinical Pharmacy, School of Pharmacy, Taipei Medical University, Taipei, Taiwan; 2 Department of Clinical Pharmacy, Master Program for Clinical Pharmacogenomics and Pharmacoproteomics, School of Pharmacy, Taipei Medical University, Taipei, Taiwan; 3 Department of Pharmacy, Wan Fang Hospital, Taipei Medical University, Taipei, Taiwan; 4 Cancer Center, Kaohsiung Medical University Hospital, Kaohsiung, Taiwan; 5 Center for Biomarkers and Biotech Drugs, Kaohsiung Medical University, Kaohsiung, Taiwan; 6 Division of Colorectal Surgery, Department of Surgery, Kaohsiung Medical University Hospital, Kaohsiung Medical University, Kaohsiung, Taiwan; 7 Graduate Institute of Clinical Medicine, College of Medicine, Kaohsiung Medical University, Kaohsiung, Taiwan; 8 Department of Surgery, Faculty of Medicine, College of Medicine, Kaohsiung Medical University, Kaohsiung, Taiwan; 9 Department of Health Industry Management, School of Health Care Management, Kainan University, Taoyuan, Taiwan

## Abstract

*Helicobacter pylori* has been identified as one of the major causes of chronic gastritis, gastric and duodenal ulcers, and gastric cancer. Lipopolysaccharide (LPS) is a major component of the outer membrane of gram-negative bacteria, and *H*. *pylori* LPS might play an exclusively important role in activating inflammatory pathways in monocytes and macrophages. To study the role of LPS in the underlying mechanism of inflammatory responses, we established an *in vitro* model using the human AGS gastric cancer cell line. We found that LPS mediates inflammation through setting off a cascade of events: activation of the store-operated calcium (SOC) channel, initiation of downstream NF-κB signaling, and phosphorylation of extracellular signal-regulated kinase 1/2 (ERK1/2). Phosphorylated ERK1/2 promotes the nuclear translocation of NF-κB, and eventually elevates the expression level of *COX-2*, a major inflammatory gene.

## Introduction

Gastric cancer (GC) is one of the most common cancers in the world and ranks second in overall cancer-related deaths^1,2^. Several major factors are known to increase the risk of developing GC, such as infection by *Helicobacter pylori* and Epstein-Barr virus, tobacco use, diet, lifestyle, and obesity. Interestingly, inflammatory responses are a common underlying mechanism shared by many of the above-mentioned risk factors^3,4^. The incidence of GC, which can span several decades, is characterized by its slow, gradual evolution. In chronological order, it gradually develops from superficial gastritis to glandular atrophy, intestinal metaplasia, dysplasia, and finally, adenocarcinoma^5^. Mounting scientific evidence led to the classification of *H*. *pylori* as a group I carcinogen for GC by the International Agency for Research on Cancer. Increasing numbers of epidemiological and animal studies have shown the causal relationship between *H*. *pylori* infection and GC^4–7^. It is well-established that *H*. *pylori* causes infection-initiated chronic gastritis, and has been thoroughly characterized by its various inflammation-triggering cellular components, including flagella; lipopolysaccharide (LPS); vacuolating cytotoxin (VacA); cytotoxin-associated gene pathogenicity islands (cagPAIs); the effector protein, CagA; peptidoglycan; glutamyl transpeptidase (GGT); protease HtrA; adhesins BabA and SabA; and others^4,8–10^.

LPS, known as endotoxin, displays robust immunostimulatory abilities upon recognition by toll-like receptor 4/MD-2^11^. LPS is composed of a glycolipid terminal structure termed the lipid A-core, which is mainly responsible for the endotoxicity of LPS, and an O-antigen polysaccharide^12^. Cytokine induction assay performed by other research groups suggested that the structures of *H*. *pylori* LPS and lipid A can modulate immune responses during infection, and both play roles in chronic inflammatory responses^13–16^.

There are two common isoforms of cyclooxygenase (COX), namely COX-1 and COX-2, and COX-1 is known as the constitutively expressed isoform. In humans, COX-1 and prostaglandin synthesis are indispensable to some essential physiological processes, such as stomach mucosa maintenance, platelet function, blood vessel protection, and regulation of renal blood flow pressure^17–20^. COX-2 is an inducible enzyme that plays a key role in the synthesis of prostaglandins in response to inflammatory stimuli. In addition, *COX-2* gene expression was also found to respond to other stimuli, including growth factors, endotoxin, carcinogen, hormones, and chemokines^21^. According to previous studies on various types of cancer, such as esophageal cancer, GC, colorectal cancer, etc., overexpression of *COX-2* in cancerous tissue was observed^22–24^. Collective studies also revealed the correlation between *COX-2* overexpression and decreased survival rates in cancer patients, and some have described the association of prostaglandin, a downstream product of *COX-2*, with tumor angiogenesis^25,26^. In this paper, we report on interactions and the hierarchy of elements involved in *COX-2* gene activation in AGS cells. Our results substantiate that the store-operated calcium (SOC) channel, extracellular signal-regulated kinase (ERK), and nuclear factor kappa B (NF-κB) are necessary mediators of LPS-induced *COX-2* gene expression in GC.

## Results

### Analysis of LPS-responsive regions in the *COX-2* promoter

**Figure 1:**
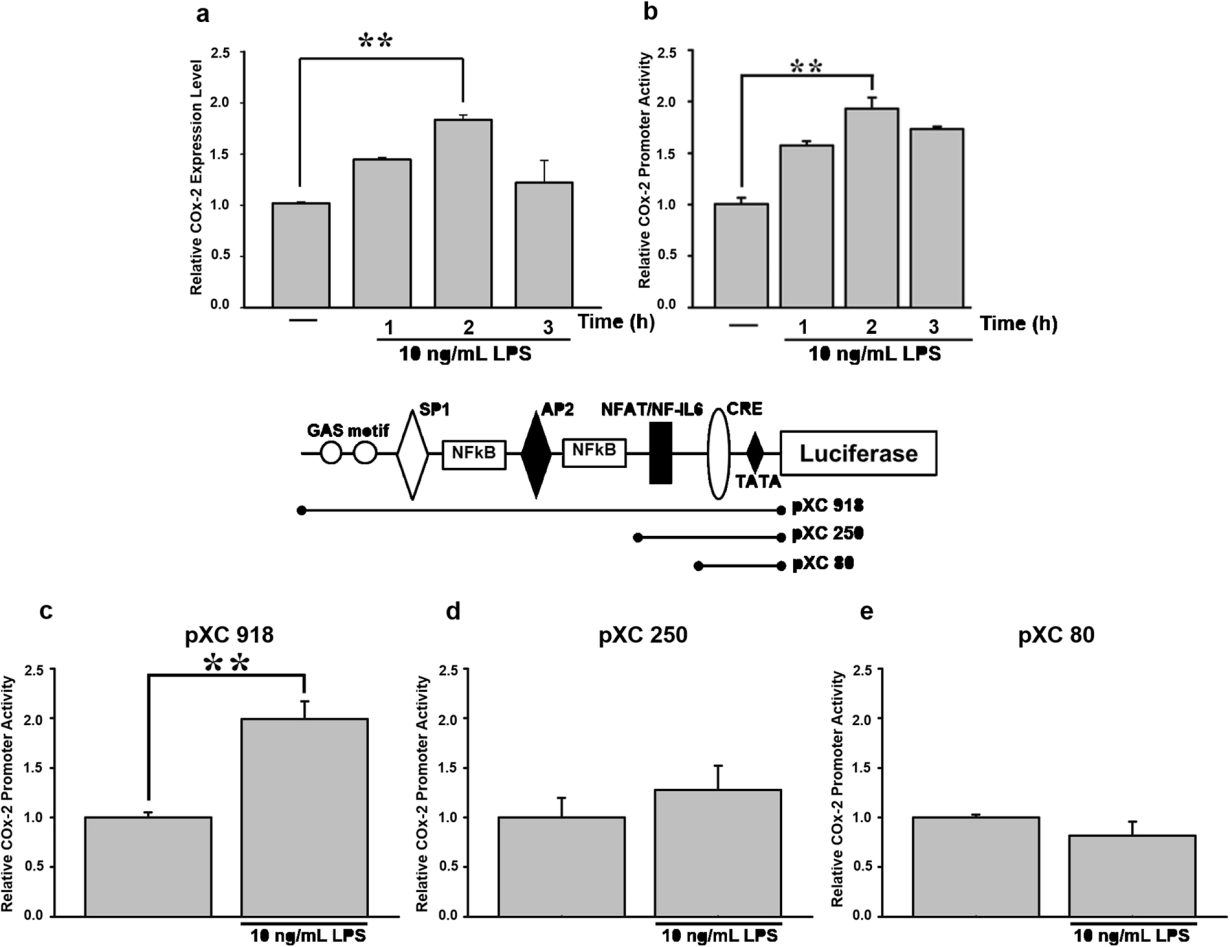
Analysis of LPS-responsive regions in the promoter area of the *COX-2* gene. (**a**) Cells were treated with or without 10 ng/mL LPS for 1, 2, and 3 h. Total RNA was extracted from AGS cells to quantify *COX-2* gene expression by real-time PCR. (**b**) Cells were transfected with 0.5 μg of a *COX-2* promoter-driven luciferase reporter construct (pXC 918) for 24 h, and then treated with or without 10 ng/mL LPS for 1, 2, and 3 h. *COX-2* promoter activity was measured with luciferase assay. Various lengths of the *COX-2* promoter of (**c**) pXC 918 (full length), (**d**) pXC 250, and (**e**) pXC 80 were respectively transfected into AGS cells. After 24 h, cells were incubated with 10 ng/mL LPS for 2 h. Promoter activity was measured with luciferase assay. Values for luciferase activity are presented as the mean ± SEM. Statistical significance (**p < 0.01) of the difference between control and LPS-treated cells was determined by Student’s *t*-test.

To determine the role of LPS in *COX-2* gene regulation, AGS cells were treated with 10 ng/mL LPS. *COX-2* gene activity peaked at 2 h post-treatment in both the real-time PCR and luciferase reporter assay (Fig. 1a,b). A *COX-2* promoter-driven luciferase reporter plasmid, pXC918, was used in the reporter assay. pXC918, pXC250, and pXC80, plasmids containing different fragments of the *COX-2* promoter (respectively −918, −250, −80 bp upstream of the *COX-2* gene), were used to locate potential responsive elements in the *COX-2* promoter. A two-fold increase in promoter activity of pXC918 (which contained Sp1, AP2, NF-κB, NFAT-binding sites, and the cyclic AMP response element (CRE) motif^27^ was recorded after transfected AGS cells were incubated with 10 ng/mL LPS (Fig. 1c). However, pXC250 (which contained the NFAT-binding site and CRE) and pXC80 (which contained only the CRE) did not show a similar increase in promoter activity (Fig. 1d,e).

### Inhibition of LPS-induced NF-κB activation attenuates *COX-2* expression

**Figure 2:**
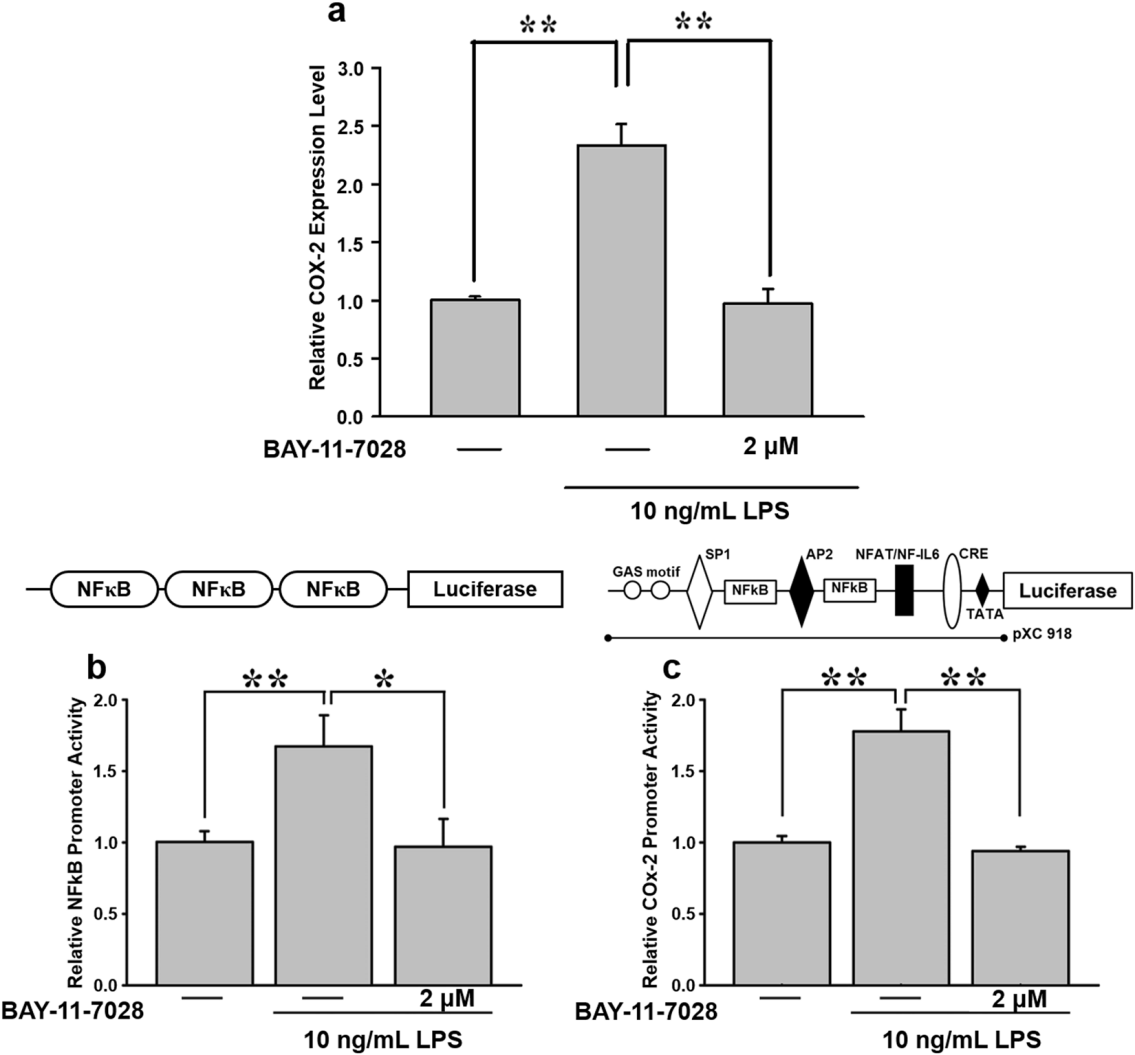
Effects of BAY 11-7082, a NF-κB inhibitor, on LPS-mediated *COX-2* expression in AGS cells. (**a**) Cells were pre-treated with 2 μM BAY 11-7082 for 30 min, and then stimulated with 10 ng/mL LPS for 2 h. Total RNA was extracted from AGS cells for *COX-2* gene detection using real-time PCR. To further investigate the effect of BAY 11-7082 on LPS-mediated *COX-2*, cells were transiently transfected with 0.5 μg of (**b**) a luciferase reporter construct with the 3X NF-κB binding motif and (**c**) a *COX-2* promoter (pXC918). After 24 h, cells were pre-incubated with 2 μM BAY 11-7082 for 30 min, followed by 10 ng/mL LPS for 2 h. Statistical significance (*p < 0.05, **p < 0.01) of the difference between control and LPS-treated cells was determined by Student’s *t*-test.

NF-κB is a transcription factor well known for regulating inflammatory and immune reactions. To determine the role of NF-κB in LPS-induced *COX-2* expression, the NF-κB inhibitor, BAY 11-7082 (2 μM), was used. As shown in Fig. 2a, BAY 11-7082 suppressed LPS-induced *COX-2* messenger (m)RNA expression. Furthermore, the luciferase assay showed that pretreatment with BAY 11-7082 abolished both NF-κB reporter activity (Fig. 2b) and *COX-2* promoter activity (Fig. 2c). These results imply a role of NF-κB in LPS-mediated *COX-2* expression in AGS cells.

### LPS promotes ERK1/2 phosphorylation in a time-dependent manner

**Figure 3:**
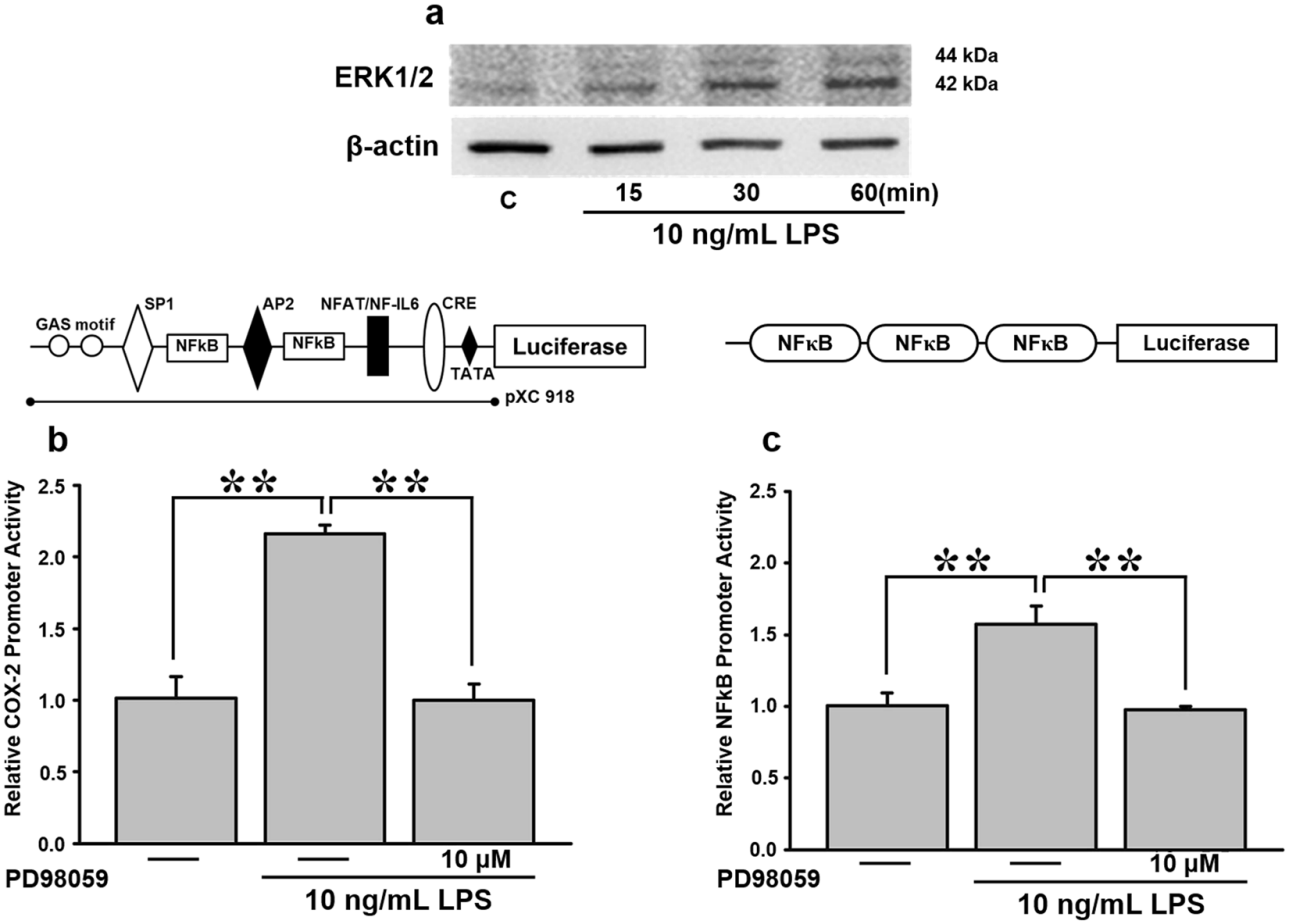
Effects of PD98059, a pERK1/2 inhibitor, on LPS-mediated *COX-2* expression in AGS cells. (**a**) Cells were treated with and without 10 ng/mL LPS for 15, 30, and 60 min. Cell lysates were harvested for a pERK1/2 expression analysis. To further determine the effect of PD98059 on LPS-medicated *COX-2*, cells were transiently transfected with 0.5 μg of (**b**) a *COX-2* promoter reporter construct (pXC918) and (**c**) 3X NF-κB reporter construct for 24 h. Cells were incubated with 10 μM PD98059 for 30 min, and stimulated with 10 ng/mL LPS for 2 h. Promoter activity was measured with a luciferase assay. Statistical significance (**p < 0.01) of the difference between control and LPS-treated cells was determined by Student’s *t*-test.

ERK phosphorylation is crucial for the activation and nuclear translocation of NF-κB. Thus, we investigated the connection between LPS and ERK1/2, and its effect on *COX-2* expression. Immunoblot results showed that pERK1/2 significantly increased in a time-dependent manner when cells were treated with LPS (Fig. 3a). Next, to clarify the connection between LPS-induced ERK1/2 phosphorylation and *COX-2* expression, the *COX-2* promoter-driven luciferase reporter, pXC918, was transfected into cells. At 24 h post-transfection, cells were incubated with 10 ng/mL LPS for 2 h, and 10 μM PD98059, a pERK1/2 inhibitor, for 30 min. We found that PD98059 effectively abolished LPS-induced *COX-2* promoter activity down to the basal level (Fig. 3b).

Previous studies suggested that ERK phosphorylation is responsible for the increase in NF-κB activity^28^. To further confirm whether LPS-induced ERK phosphorylation mediates downstream NF-κB activity, AGS cells transfected with the NF-κB luciferase reporter were pretreated with the pERK1/2 inhibitor, PD98059 (10 μM), for 30 min, followed by 10 ng/mL LPS for 2 h. The luciferase assay demonstrated that PD98059 inhibition coincided with reduced NF-κB activity, and thus confirmed the relation between ERK and NF-κB, and their relative positions in the signaling cascade (Fig. 3c).

### Effects of SOC channels inhibitor in LPS-induced *COX-2* gene expression

**Figure 4:**
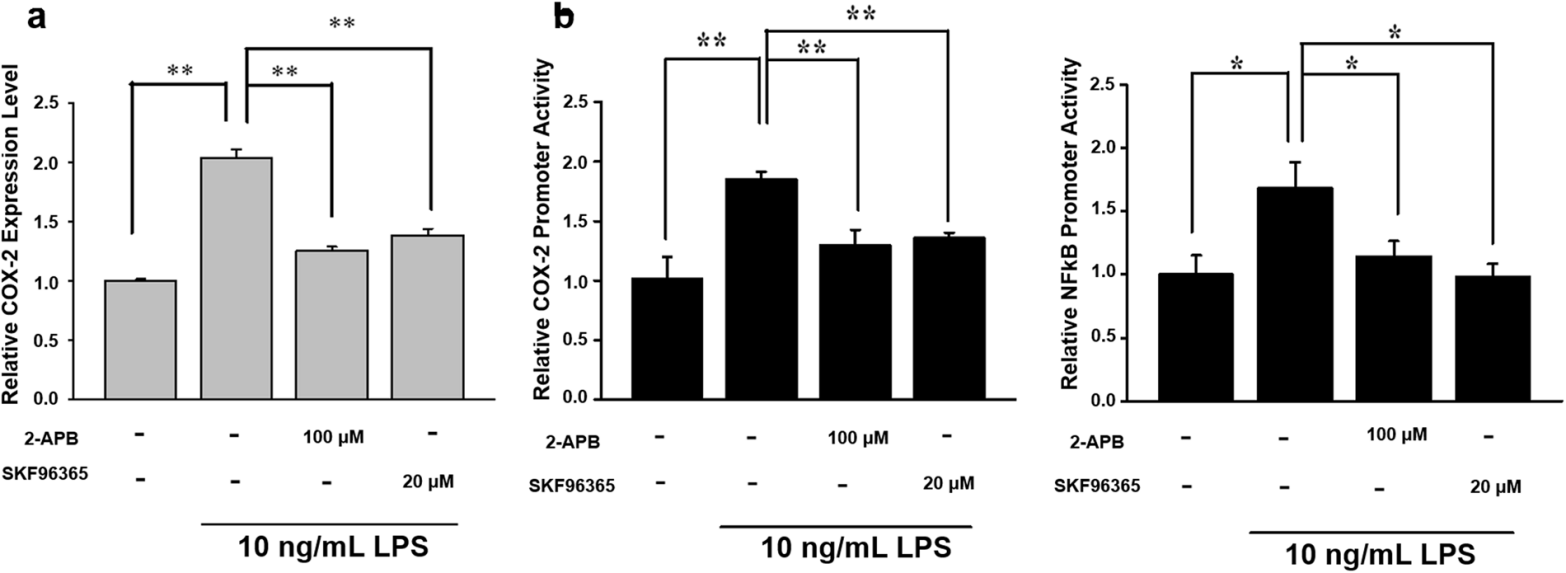
Effects of store-operated Ca^2+^ (SOC) channel inhibitors on LPS-induced *COX-2* gene expression. (**a**) Cells were pretreated with SOC channel inhibitors, i.e., 100 μM 2-APB and 20 μM SKF96365 for 30 min, followed by 10 ng/mL LPS for 2 h. Total RNA was extracted from AGS cells to quantify *COX-2* gene expression using a real-time PCR. (**b**) A *COX-2* promoter reporter construct (pXC918) and NF-κB reporter were transfected into cells. Transfected cells were treated with 100 μM 2-APB or 20 μM SKF96365, followed by 10 ng/mL LPS. Luciferase activity was measured and represented in fold change of promoter activity and was normalized to basal activity. Statistical significance (*p < 0.05, **p < 0.01) of the difference was determined by Student’s *t*-test.

Our previous study showed that reduced SOC influx attenuates lung cancer cell proliferation via ERK phosphorylation^29^. Studies by other groups also suggested that the SOC channel and cytosolic calcium are correlated with ERK1/2 phosphorylation^30,31^. In addition, activation of NF-κB is reported to be directly associated with the cytosolic calcium level and calcium-related signaling under various circumstances^32–34^. Hence, we investigated whether the SOC channel plays a role in ERK phosphorylation and NF-κB translocation, and eventually leads to an increase in *COX-2* gene expression. AGS cells were pretreated with the SOC channel inhibitor, 2-APB (100 μM) or SKF96365 (20 μM), followed by 10 ng/mL LPS stimulation for 2 h. A real-time PCR was used to quantify *COX-2* expression, and results showed that SOC channel inhibition abolished LPS-induced *COX-2* gene expression (Fig. 4a). Moreover, SOC channel inhibitors also demonstrated the ability to suppress both the *COX-2* promoter and NF-κB reporter activity (Fig. 4b). These results support the fact that the SOC channel is an upstream regulator of NF-κB in LPS-induced *COX-2* expression.

### Blockade of LPS-induced calcium influx by SOC channel blockers and calcium chelators

**Figure 5:**
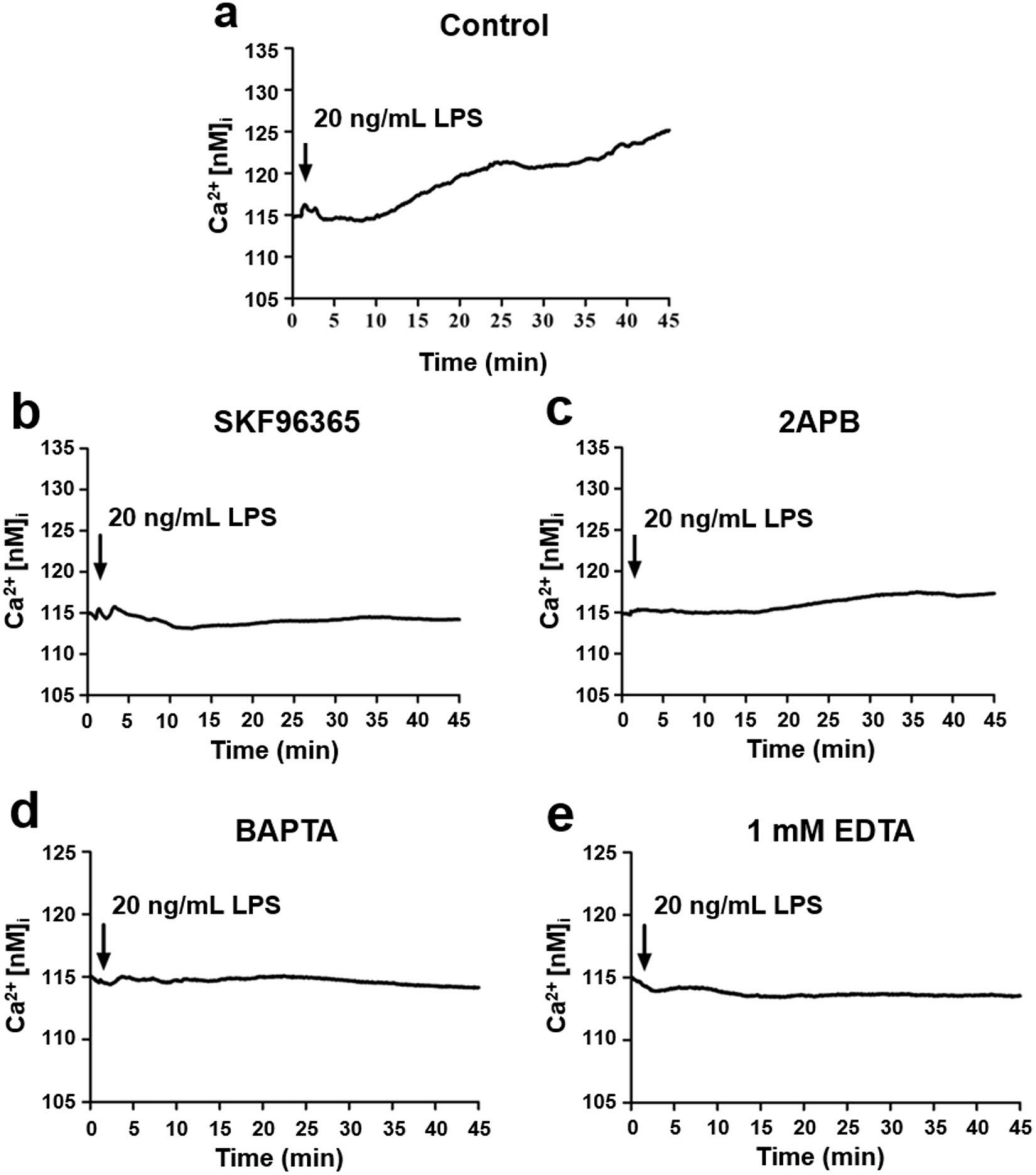
Blockade of LPS-induced calcium influx by store-operated calcium (SOC) channel inhibitors and calcium chelators. Cellular calcium influx of AGS cells treated without (**a**) or with 20 μM SKF96365 (**b**), 100 μM 2APB (**c**), 5 μM BAPTA (**d**), or 1 mM EDTA (**e**) prior to subsequent intracellular calcium imaging was induced by 20 ng/mL LPS in 2 mM calcium buffer and detected by fluorescence microscopy for up to 45 min. The intracellular calcium concentration was estimated by measuring the signal of Fluo-4, a calcium indicator, inside AGS cells.

AGS cells were respectively treated with 20 μM SKF96365, 100 μM 2APB, 5 μM BAPTA, and 1 mM EDTA, followed by 20 ng/mL LPS (Fig. 5a). The intracellular calcium concentration was estimated by measuring the Fluo-4 signal. LPS-induced SOC entry (SOCE) was effectively blocked by SOC channel-specific inhibitors (Fig. 5b,c), as well as calcium chelators (Fig. 5d,e). These results suggest that LPS triggers the SOCE, and causes downstream signal transduction.

### Identification of the role of SOC channel in LPS-induced *COX-2* gene expression

**Figure 6:**
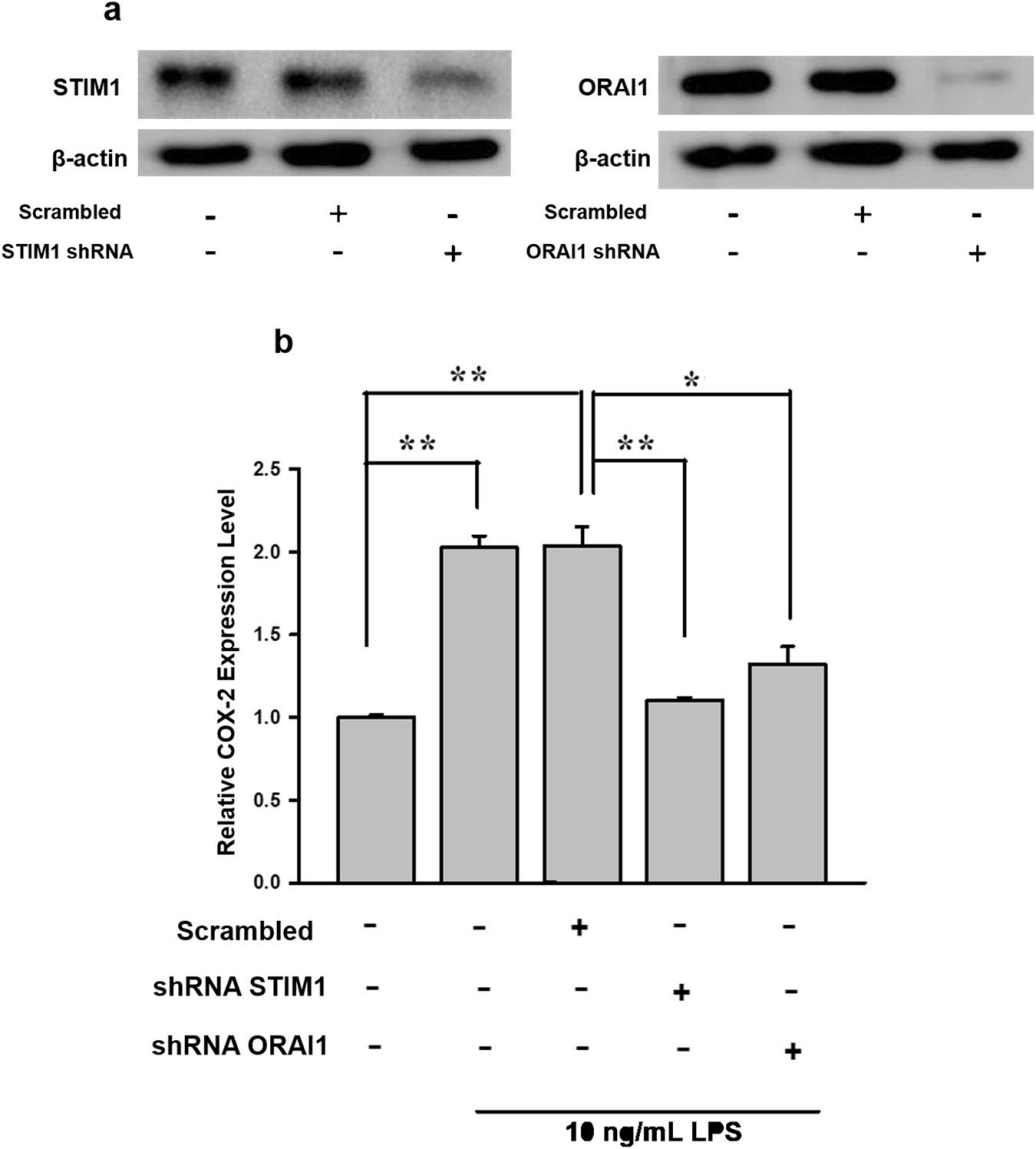
The roles of STIM1 and ORAI1 in LPS-induced *COX-2* gene expression. *STIM1* shRNA and *ORAI1* shRNA were respectively transfected into AGS cells using Lipofectamine. (**a**) Immunoblot of STIM1 (80 kDa) and ORAI1 (31 kDa) after knockdown of the respective gene. 2 μg shRNA was used for transfection, and the total cell lysate was harvested for immunoblotting at 24 h post-transfection. (**b**) Cells were treated either with or without LPS after knockdown of *STIM1* or *ORAI1*. RNA was extracted for *COX-2* mRNA quantification by a real-time PCR. Statistical significance (*p < 0.05, **p < 0.01) of the difference was determined by Student’s *t*-test.

To validate the effects of SOC channel inhibitors (2-APB and SKF96365), AGS cells were transfected with *STIM1* or *ORAI1* short hairpin (sh)RNA to observe the effect of knockdown of the SOC channel on LPS-induced *COX-2* gene expression. As shown in Fig. 6a, 2 µg *STIM1* shRNA or *ORAI1* shRNA was used, and the respective gene was significantly suppressed. Furthermore, we found that silencing of either *STIM1* or *ORAI1* significantly impaired LPS-induced *COX-2* gene expression (Fig. 6b). The data sufficiently demonstrated that calcium influx via STIM1/ORAI1-mediated SOCE is crucial for LPS-induced *COX-2* expression.

## Discussion

*Helicobacter pylori*, a gram-negative spirochete, infects more than half of the world’s population^35^. Typically, *H*. *pylori* infections are more widespread in developing countries, which are often plagued by water pollution and unsanitary living conditions. After decades of rigorous study and data accumulation, many researchers support the notion that *H*. *pylori* infection is the trigger of intestinal gastric adenocarcinoma^2,36^. Through a variety of mechanisms, *H*. *pylori* infection can amount to detrimental changes in gastric epithelial cells, and eventually the occurrence of cancer^4,37,38^. The pathogenicity of *H*. *pylori* is mainly associated with various bacterial cell components, including flagella, vacuolating toxin (VacA), cytotoxin-associated gene pathogenicity islands (cagPAIs), and lipopolysaccharide (LPS)^10^. Moreover, reports suggest that LPS from *H*. *pylori* increases the paracellular permeability of gastric cells, which can compromise the stomach’s mucosal defense and increase susceptibility to developing medical conditions^39,40^. LPS was also shown to upregulate vascular endothelial growth factor (VEGF) and *COX-2*, which contributed to failure of ulcer healing in a rat stomach ulcer model^41^.

*COX-2* is known to promote tumorigenesis through prostanoid biosynthesis, which includes prostaglandin E2 (PGE2). Inhibition of *COX-2* and PGE2 receptor signaling leads to suppression of tumor development in a variety of animal models^42,43^. Our results are consistent with a previous study by Franchi *et al*., which showed significantly elevated *COX-2* and PGE2 levels in the lungs and stomach of LPS-treated rats compared to controls^44^. The *COX-2* gene promoter contains a considerable number of transcription factor-binding motifs. Several studies established that activator protein 1^45^, CCAAT/enhancer-binding protein β^46^, cyclic AMP-responsive element-binding protein^47^, and NF-κB^48^ are transcriptional regulators of *COX-2* ^49–51^. Our results suggest that the LPS-responsive element in the *COX-2* promoter is located in the -918 to -250 region, which includes AP-2, SP-1, and two NF-κB-binding motifs (Fig. 1). The transcription factor, NF-κB, is one of the primary mediators of immune and inflammatory responses, in addition to its roles in critical cellular processes during carcinogenesis, such as transformation, proliferation, angiogenesis, and metastasis^52^. NF-κB activation was shown to be modulated by numerous proinflammatory stimuli, ranging from membrane toll-like receptor (TLR) activation by pathogens to specific cytokines (paracrine or endocrine), through both canonical and non-canonical pathways^53^. Due to its roles in inflammation and immunity, NF-κB signaling and its activation by *H*. *pylori* have piqued the interest of many researchers.

**Figure 7:**
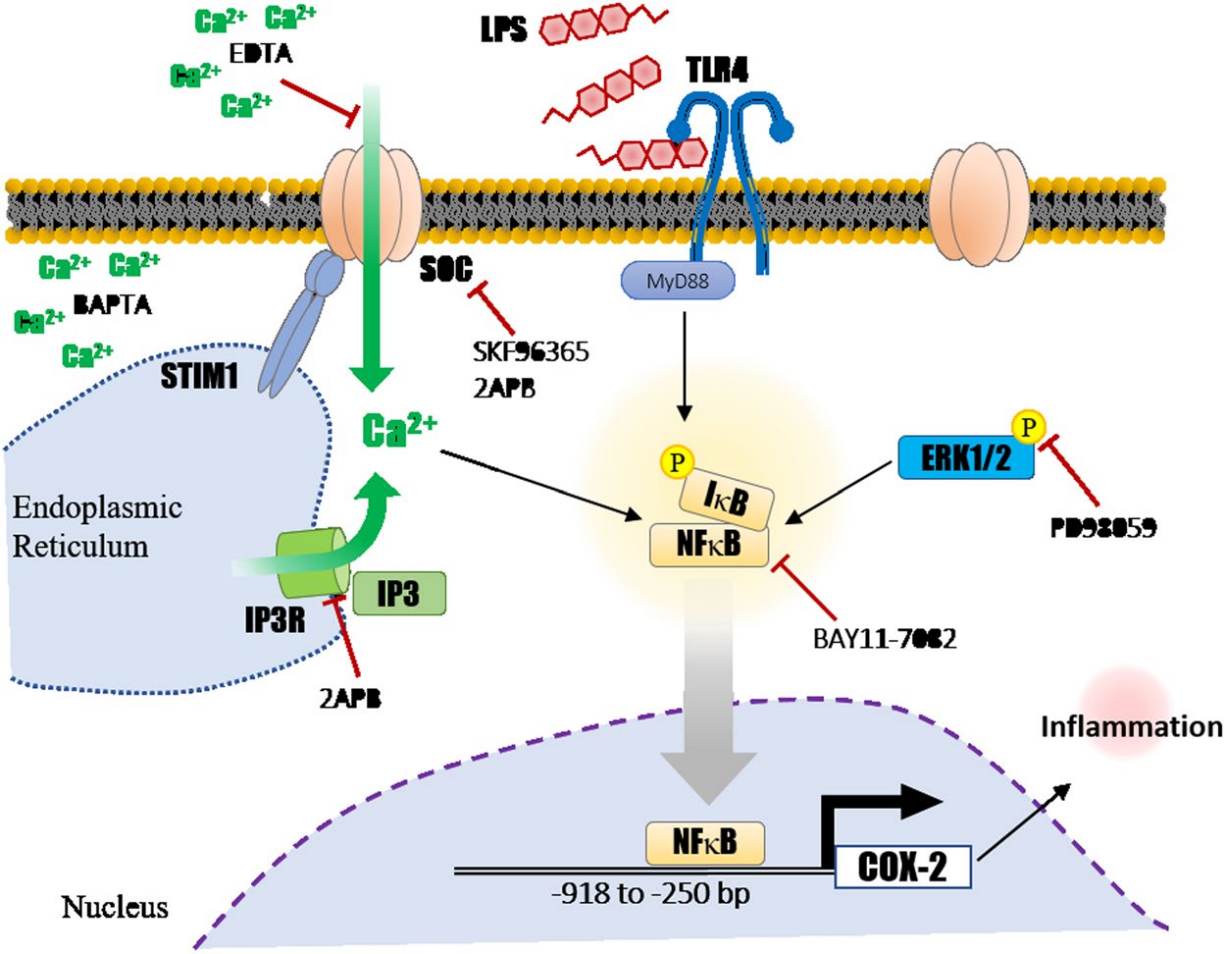
Schematic representation showing a preliminary model of LPS-induced *COX-2* gene activation in AGS cells.

Our work demonstrates that LPS might rely on the activation of SOC channel and calcium influx to promote the phosphorylation of ERK, and subsequently, the nuclear translocation of NF-κB. Upon nuclear entry, NF-κB acts as a transcription factor, and drives *COX-2* expression. A schematic representation of our proposed LPS-induced *COX-2* activation is depicted in Fig. 7. It is well-established that SOCE is essential for T cell maturation, mast cell degranulation, and many other calcium-dependent cellular processes. Our recent study revealed a role for SOCE in cancer invasion and metastasis in colorectal cancer patients, where relatively high expression levels of the calcium storage sensor, STIM1, were observed in colorectal cancer tissues^51^. Furthermore, STIM1 expression dynamics were positively correlated with increased malignancy^54^. In the lung cancer cells, previous studies also indicated that store-operated calcium entry is involved in *COX-2* gene activation as well as cell cycle progression^29,49^ We suspect that after the cell’s initial exposure to LPS, inositol 1,4,5-trisphosphate receptor (IP_3_R) was activated by IP_3_, and quickly depleted ER calcium store. Upon sensing the depletion, STIM1 subsequently binds to ORAI1 and activates calcium intake via membrane-bound SOC channels. This study implies yet another role for SOCE in LPS-induced *COX-2* expression, as well as the inflammatory response in GC cells.

The *in situ* expression and biological functions of COX isoforms in various tissues are well characterized. Studies suggest that the expression and function of the *COX* gene may vary under different circumstances. For example, *COX-2* is constitutively expressed in the spine, instead of the usual *COX-1* isoform, and is responsible for hyperalgesia during injury^55^. Thus, it is important to recognize that the effects of LPS and the signaling pathway described in this study are probably highly contextual and tissue specific. Despite the rather clear outline, our findings have yet to completely rule out participation by other alternative pathways in LPS-induced *COX-2* expression, especially since *COX-2* is noted for responding to multiple stimuli. Although *COX-2* is more commonly known for its role in pro-inflammatory contexts, its dual regulatory roles in production of pro-inflammatory prostaglandins and anti-inflammatory prostaglandins need to be further addressed when studying inflammation-related conditions, such as gastritis in our case^56^. Hence, profiling relevant types of prostaglandins synthesized in gastric cells at each stage of inflammation can be crucial for the understanding of induced *COX-2* at protein level and subsequent downstream events. Hopefully, knowledge gained from a better understanding of the underlying molecular network of LPS-induced *COX-2* expression can potentially serve as the basis of novel therapeutic approaches for gastritis and GC in the future.

## Materials and Methods

### Cell culture

Human stomach adenocarcinoma AGS cells were purchased from American Type Culture Collection (ATCC, Manassas, VA, USA). Cells were cultured in Dulbecco’s modified Eagle’s medium (DMEM) (Invitrogen, Carlsbad, CA, USA) supplemented with 10% fetal bovine serum (FBS; Invitrogen) and 1% penicillin-streptomycin (Invitrogen), and cultured at 37 °C in a 5% CO_2_ atmosphere. In these series of experiments, cells were treated with 10 ng/mL LPS in DMEM.

### RNA isolation and a quantitative real-time reverse-transcription polymerase chain reaction (RT-qPCR)

Total RNA was isolated from AGS cells with Trizol reagent (Invitrogen). Complementary (c)DNA was synthesized from 1 μg of total RNA using reverse transcription kit (Invitrogen) according to manufacturer’s instructions. cDNA was diluted 1:30 with PCR-grade water and then stored at −20 °C. Gene expression levels were quantified with the Applied Biosystems StepOnePlus™ System (Thermo Fisher Scientific) with pre-optimized conditions. Each PCR was performed in triplicate using 5 μL of 2x SYBR Green PCR Master Mix, 0.2 μL of primer sets, 1 μL cDNA, and 3.6 μL nucleotide-free H_2_O to yield 10 μL per reaction. Expression rates were calculated as the normalized CT difference between the control and sample after adjusting for the amplification efficiency relative to the expression level of the housekeeping gene, β-actin. *COX-2* primers (sense, CCCTTGGGTGTCAAAGGTAA, and antisense, GCCCTCGCTTATGATCTGTC) and β-actin primers (sense, ATCTCCTTCTGCATCCTGTCGGCAAT, and antisense, CATGGAGTCCTGGCATCCACGAAAC) were used in the PCR.

### Cell transfection and luciferase assay

Lipofectamine 2000 (Invitrogen) and Opti-MEM medium (Invitrogen) were used to deliver the plasmid into AGS cells. The transfection procedure was performed according to instructions of the manufacturer. Cells were briefly treated with LPS after overnight transfection, and then lysed with cell lysis buffer. A dual-luciferase reporter assay kit (Promega, Madison, WI, USA) was used to measure the luciferase activity of the NF-κB reporter and *COX-2* promoter. *STIM1* and *ORAI1* shRNA constructs were obtained from RNAi core of Academia Sinica, Taipei, Taiwan.

### Intracellular calcium imaging

AGS cells grown in 10%-FBS DMEM were trypsinized, seeded onto 20-mm glass coverslips in a 6-well plate, and incubated for 24~48 h at 37 °C. Cells were washed with a 2 mM calcium solution and incubated with 1 μM Fluo-4 AM (Molecular Probes, Eugene, OR, USA) plus treatment with or without calcium channel blockers or calcium chelators for 30 min at 37 °C prior to intracellular calcium detection. Following treatment and staining steps, the calcium influx of AGS cells was assessed by measuring the fluorescent intensity of the intracellular calcium signal as described in our previous study [PMID: 23774942]. Cells were maintained in 2 mM calcium buffer and stimulated by 20 ng/mL LPS for up to 45 min.

### Western blotting

Protein samples (60 μg) were heated to 95 °C for 5 min and loaded onto 10% sodium dodecyl polyacrylamide gel electrophoresis (SDS-PAGE). Proteins were transferred to polyvinylidene difluoride (PVDF) membranes and blocked with 5% nonfat dry milk for 1 h at room temperature. Membranes were washed with 0.1% PBST (phosphate-buffered saline (PBS) and Tween20) three times and then incubated with primary antibodies overnight at 4 °C. Antibodies against pERK1/2 (Cell Signaling, Beverly, MA), STIM1 (Cell Signaling, Beverly, MA), and ORAI1 (GeneTex, Hsinchu, Taiwan) were diluted 1: 2000, whereas the antibody against β-actin was diluted 1: 10000. Membranes were then washed with 0.1% PBST three times and incubated with a 1: 5000 dilution of anti-mouse or anti-rabbit HRP-conjugated immunoglobulin G (IgG, Santa Cruz Biotechnology, Santa Cruz, CA, USA) for 1 h at room temperature. After washing with 0.1% PBST three times, signals were detected by an ECL-plus Western blotting detection system (Millipore).

### Data analysis

Statistical analyses were performed using Student’s *t*-test. A *p* value of < 0.05 was considered significant and is denoted by an asterisk (*), while a *p* value of < 0.01 is denoted by **.

### Data Availability Statement

The data that support the findings of this study are available from the corresponding author upon reasonable request.

## Electronic supplementary material

Supplementary Information
