# Supplementary material for: Store-operated Ca2+ Entry Facilitates the Lipopolysaccharide-induced Cyclooxygenase-2 Expression in Gastric Cancer Cells
Source: Sci Rep. 2017 Oct 16;7:12813. doi: 10.1038/s41598-017-12648-1 (PMC5643532; doi:10.1038/s41598-017-12648-1)
Supplement: Supplementary file 1 — Supplementary Information [file 41598_2017_12648_MOESM1_ESM.pdf]

Store-operated Ca^{2+} Entry Facilitates the Lipopolysaccharide-induced Cyclooxygenase-2 Expression in Gastric Cancer Cells

Jhen-Hong Wong[a#], Kuo-Hao Ho[a#], Sean Nam[a#], Wen-Li Hsu[a], Chia-Hsien Lin[i], Che-Mai Chang[a], Jaw-Yuan Wang[e,f,g,h]*, Wei-Chiao Chang[a,b,c,d,e]*

Supplementary Information

M C 15 30 60 min (10 ng/mL LPS)

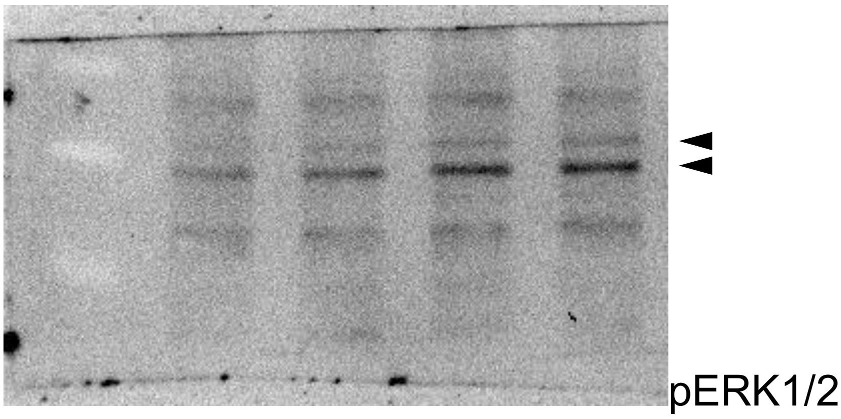

pERK1/2

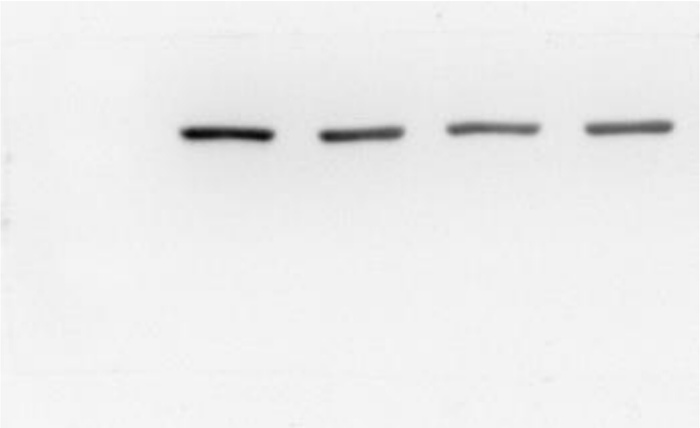

ACTB (pERK)

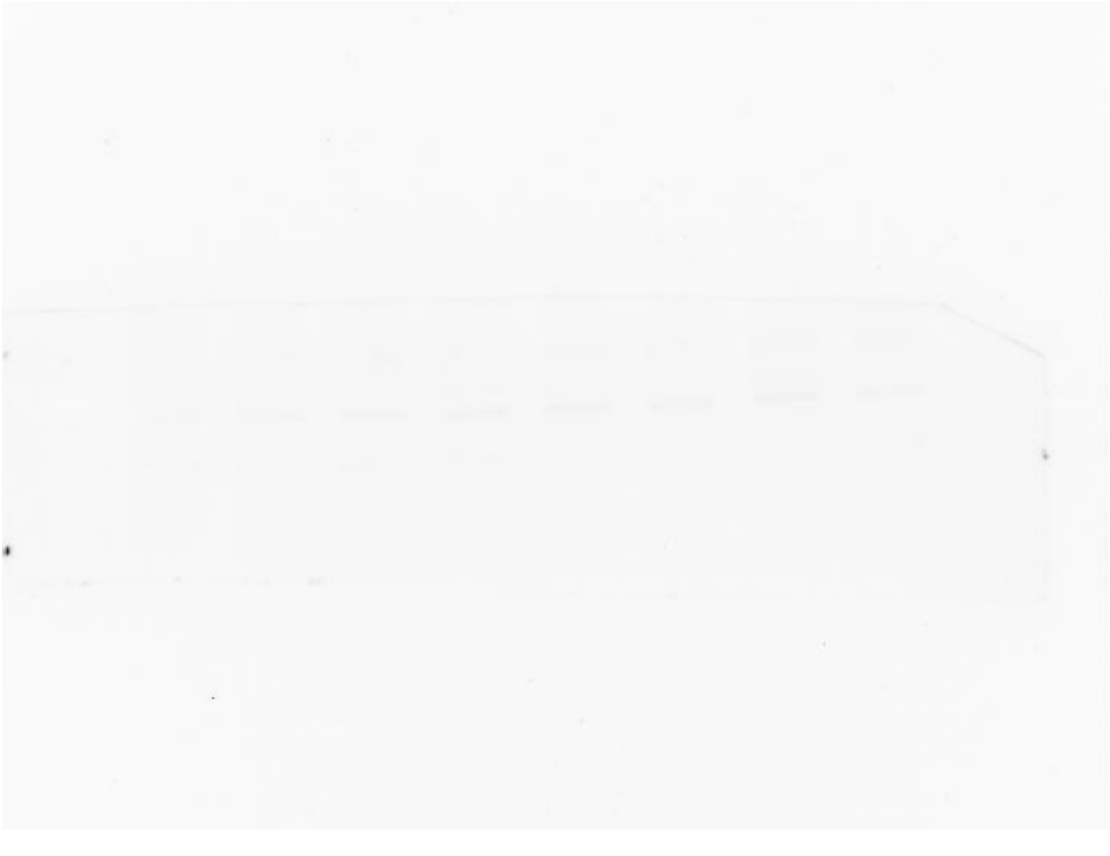

pERK

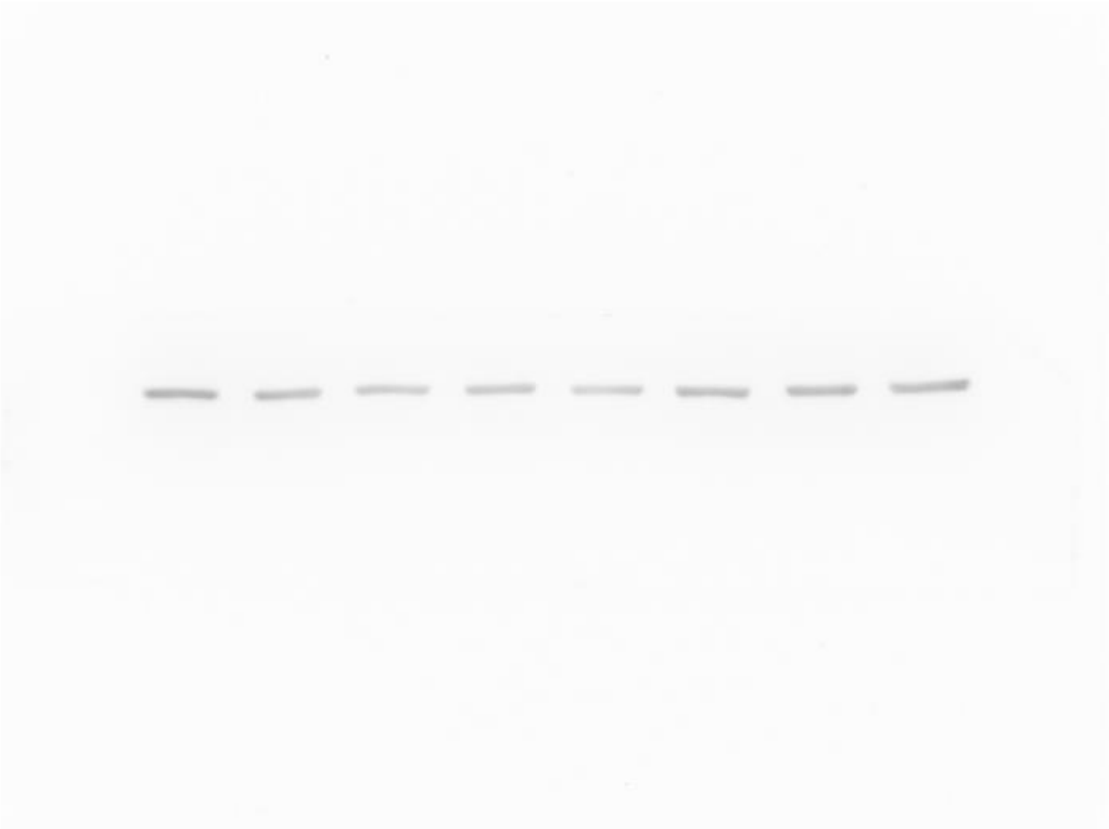

ACTB

Figure S1 Immunoblot of LPS treatment time course and pERK1/2 activation

M (-) Scr KD

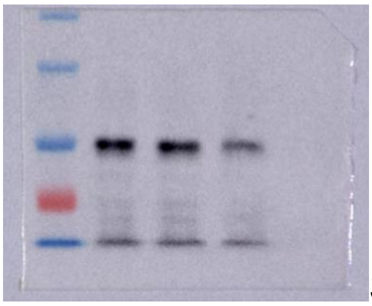

STIM1

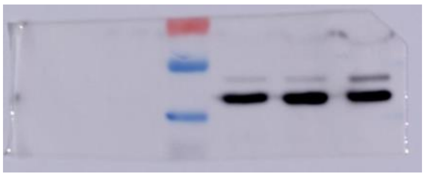

βactin(STIM1)

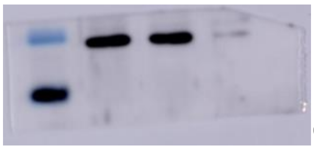

ORAI1

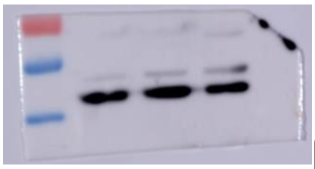

βactin(ORAI1)

Figure S2 Immunoblot of knockdown experiments of STIM1 and ORAI1. M: ladder; (-): negative control; Scr: Scrambled; KD: shRNA knockdown
